# Copper or/and arsenic induce oxidative stress-cascaded, nuclear factor kappa B-dependent inflammation and immune imbalance, trigging heat shock response in the kidney of chicken

**DOI:** 10.18632/oncotarget.21463

**Published:** 2017-10-03

**Authors:** Yu Wang, Hongjing Zhao, Yizhi Shao, Juanjuan Liu, Jinglun Li, Mingwei Xing

**Affiliations:** ^1^ Department of Physiology, College of Wildlife Resources, Northeast Forestry University, Harbin 150040, Heilongjiang, PR China

**Keywords:** copper, arsenic, oxidative stress, NF-κB, heat shock response

## Abstract

Excessive amount of copper (Cu) and inorganic arsenic (iAs) coexists in drinking water in many regions, this is associated with high risk of nephropathy, defined as chronic structural and functional disorders of the kidney. However, the underlying mechanisms are not well understood. In this study, a total of 72 day-old Hy-line chickens were exposed to 300 mg/kg copper sulphate or/and 30 mg/kg arsenic trioxide for 12 weeks. Indicators of oxidative stress, inflammation and heat shock proteins (HSPs) production were analyzed in kidney. The results showed that, when the toxicant was administrated alone, there is an antagonism between redox homeostasis during the first 4 weeks, which follows a collapse of antioxidant system manifested by damaged biomembrane structure. What's worse, oxidative damage-cascaded histopathological lesions were accompanied by increases of proinflammatory mediators and an imbalance of “Th1/Th2 drift” (Th, helper T cell) regulated by nuclear factor kappa B (NF-κB). Simultaneously, intense heat shock response went with the organism. The above-mentioned renal lesions and indicators changes were time-dependent, more complex and deteriorated effects were observed in Cu/iAs combined groups compared with the others. This study supports Cu and iAs have a synergistic type on the nephro-toxicological process additively. In conclusion, oxidative stress and inflammatory induced by Cu or/and iAs are potential mechanisms in their nephrotoxicity, increased heat shock response may play a renoprotection function in tissues damage.

## INTRODUCTION

Arsenic (As) is a ubiquitous metalloid in environment, its inorganic compounds are toxic that occur naturally in water, air, and soil then enters the food chains through geological releases, contaminated water and anthropogenic sources. One of its most abundant hazards is arsenic trioxide (As_2_O_3_), a human carcinogen which is associated with formation of tumors in skin, lung, bladder, liver and kidney [1]. Arsenism has become a major public health concern throughout the world. Copper (Cu) is an essential trace metal acting as a catalytic co-factor in an extensive of redox enzymes required metabolic processes [2]. Its deficiency may lead to several diseases in human, such as anemia, leukopenia and myeloneuropathy [3]. However, once the intake of copper exceeds the tolerable limit, it exerts toxic effects leading to cell death due to its potential to catalyze the generation of reactive oxygen species (ROS) [4]. Dating back to 1882, the accidental discovery of Bordeaux mixture and the value of copper-based preparations for the control of vine downy mildew disease, making copper used as an effective therapeutic approach for fungicides [5]. On the other hand, there are areas worldwide such as China, Europe, Australia, Bangladesh and the US facing with high concentration of arsenic from coal burning, pesticides and contaminated foodstuffs [6]. So, the situation can be alarming where there is a possibility of a section of population belonging to these areas suffering from cross contamination of copper and arsenic.

One potential mechanism by which copper and arsenic could lead to kidney injury is oxidative stress, a result of disequilibrium between free radical generation and antioxidant status, which has been implicated in several pathologies including renal failure and even uremia [7]. Oxidative stress was reported to be involved in arsenic toxicity on immune organs [8], gastrointestinal tract tissues [9] and brain tissues [10]. It is also one of the accepted mechanisms of copper toxicity, Kumar et al. reported chronic copper exposure induced high oxidative stress, thus affecting the structure and function of kidney [11]. High levels of ROS can act as mediators of inflammation, inducing peripheral inflammation [12]. This might because intracellular redox status controls nuclear factor kappa B (NF-κB) activation by regulating tyrosine phosphorylation events within the common step of the NF-κB signal transduction pathway [13]. As a manifestation of inflammation, NF-κB activation can also be suppressed by antioxidants in numerous models [14–16].

The kidney is the target organ for toxicant because of its circulation and excretion function. Under pathological conditions, secondary nephritis may contribute to degeneration and vacuolization of the tubular cells and inflammatory cell infiltration [17]. The complex inflammation is regulated by cytokine networks and evidenced by production of many pro-inflammatory indicators, which orchestrate interactions between different cells in several inflammatory diseases such as asthma and rheumatoid arthritis [18]. The control of cytokine production and cell survival in cellular responses to stimulators such as ROS, bacterial or viral antigens are caused by NF-κB, which is present in almost all animal cell types [13]. By contraries, suppression of transcriptional activities of NF-κB attenuates the release of inducible NO synthase (iNOS), cyclooxygenases-2 (COX-2), prostaglandin E2 synthases (PTGEs) and tumor necrosis factor-α (TNF-α) [19], indicating up- and downstream relationships. NF-κB is also involved in T-cell development and functional divergence, such as helper T cell (Th) 1 and Th2 differentiation [20], which are responsible for immunity against intracellular/extracellular pathogens respectively. As a hallmark mediator of Th1 cells and the signs of chronic autoimmune inflammation, production of interferon (IFN)-γ has inhibitory potential for the development of Th2 cells. Equally, as the signature cytokine of Th2 cells, interleukin (IL)-4 increases the expression of several cytokine inhibitors to downregulate the production of pro-inflammatory cytokines IL-1, IL-6, IL-8 and IL-12. All in all, Th1 and Th2 cells antagonize each other by blocking the generation of the antipodic cell type and by blocking each other's effector functions [21]. Research results indicated that the imbalance of Th1/Th2 function is associated with the pathogenesis in several nephropathy [22].

Oxidative stress induced by heavy metals can lead to a fundamental biological reaction-heat shock response [23]. Function as molecular chaperones, heat shock proteins (HSPs) were low synthesized from stress cells under stress response conditions. Under stress conditions, newly synthesized stress proteins maintain cellular homeostasis by assisting in the correct folding of nascent and stress-accumulated misfolded proteins [24]. Increasing literatures illustrate that heavy metals cause expression of heat shock proteins, such as arsenic on HSP27 and HSP70 [25], copper on HSP60, HSP70 and HSP90 [26].

During the copper smelting process, the discharges of tailings containing copper and arsenic lead to long-term accumulation through the biogeochemical circle and threating the health of animals and humans. Especially, chicken is one of the most favorite protein source for humans, which will eventually cause copper and arsenic accumulation in human body through food chain. Despite there has been quite a few studies on the toxicity of copper or arsenic, little study was reported about their combination in chicken. In this study, the levels of inflammatory mediators and HSPs were detected by qRT-PCR or Western blotting. In order to monitor renal function, the ultrastructural and histopathological changes and antioxidant status were also measured by experimental pathology and biochemistry. Therefore, in the light of perceived threat from environmental pollutants and foodstuffs, the present study was designed with an aim to assess the nephrotoxicity of copper and arsenic individual or combined exposure from the view of oxidative damage and immunity, this study will also offer basic understanding on the protective role of HSPs.

## RESULTS

### Experimental model development

From 4 weeks of the experiment, the feed intake of chickens in the 300 mg/kg CuSO_4_ or/and 30 mg/kg As_2_O_3_ groups began to decline in comparison with those of the control group. The fecal offensive odors of chickens in the treatment groups began in 6 weeks of the experiment. No mortality was found during the experiment. The results of the principal components analysis (PCA) was shown in Figure 7A, parameter determination was based on ordination plots, corresponding to the first and second principal components (PC) as 81% and 12.4%, respectively. Figure 7A clearly indicated that Cu-, As- and Cu+As-group were closest to each other at each time point in the PC1 matrix, meaning that their relationships were closer based on PC1. PCA analysis demonstrated that the effects of CuSO_4_ and As_2_O_3_ on nephrotoxicity were in a time-dependent manner, which provided reference for further research under individual or co-exposure.

### Ultrastructural and histopathological changes in kidney

**Figure 1:**
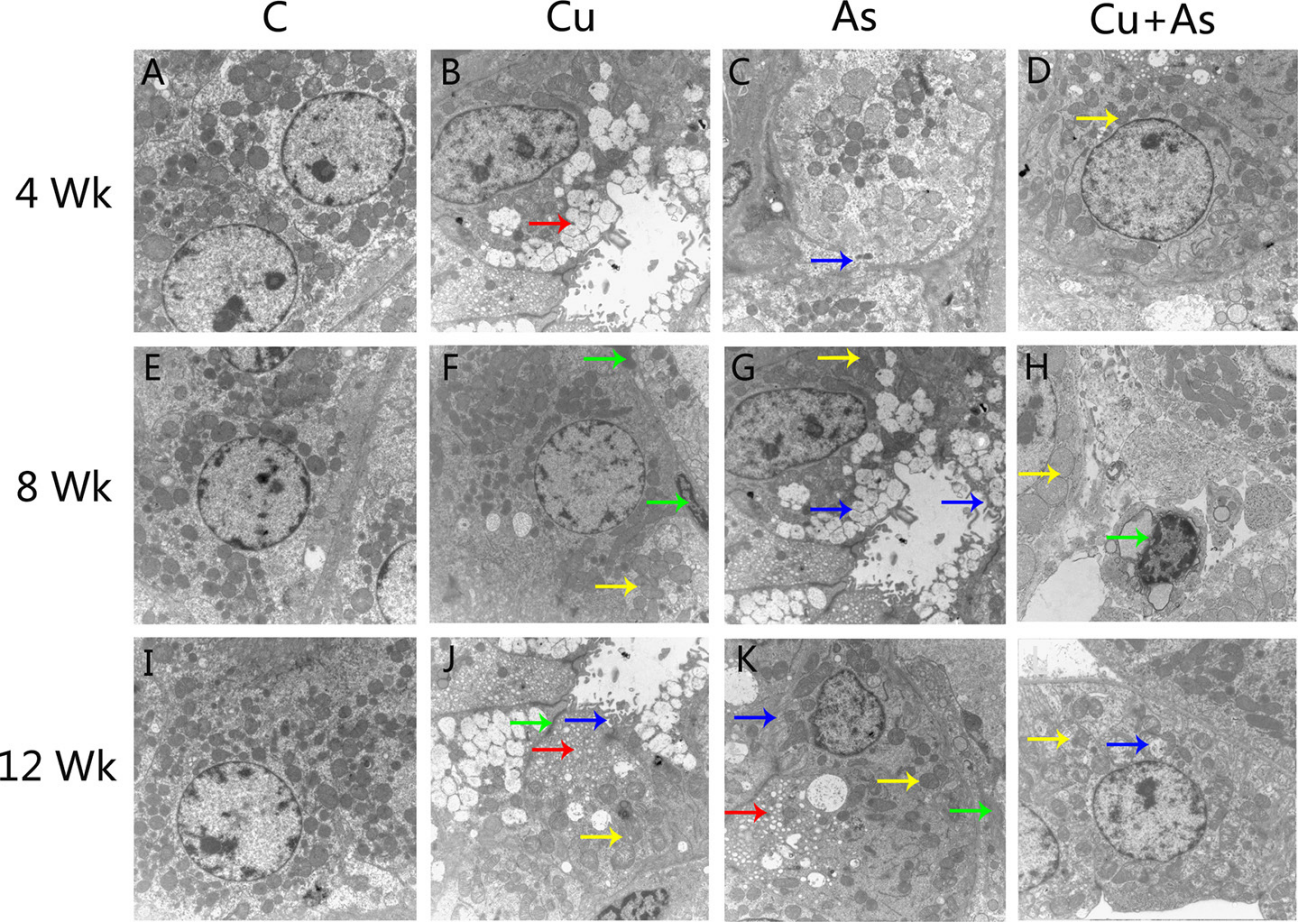
Ultrastructural changes during experiment (× 10000) Arrows: red: Vacuolization, yellow: mitochondrial swelling, blue: biomembrane damage, green: high density electron dense deposits.

As shown in Figure 1, unclear and irregular nuclear membrane, swollen mitochondria, high density electron dense deposits and cell vacuolization were observed in the experimental groups, these ultrastructural lesions induced by Cu or/and As were changed more serious compared with control group. The above lesions were not observed in the control group.

**Figure 2:**
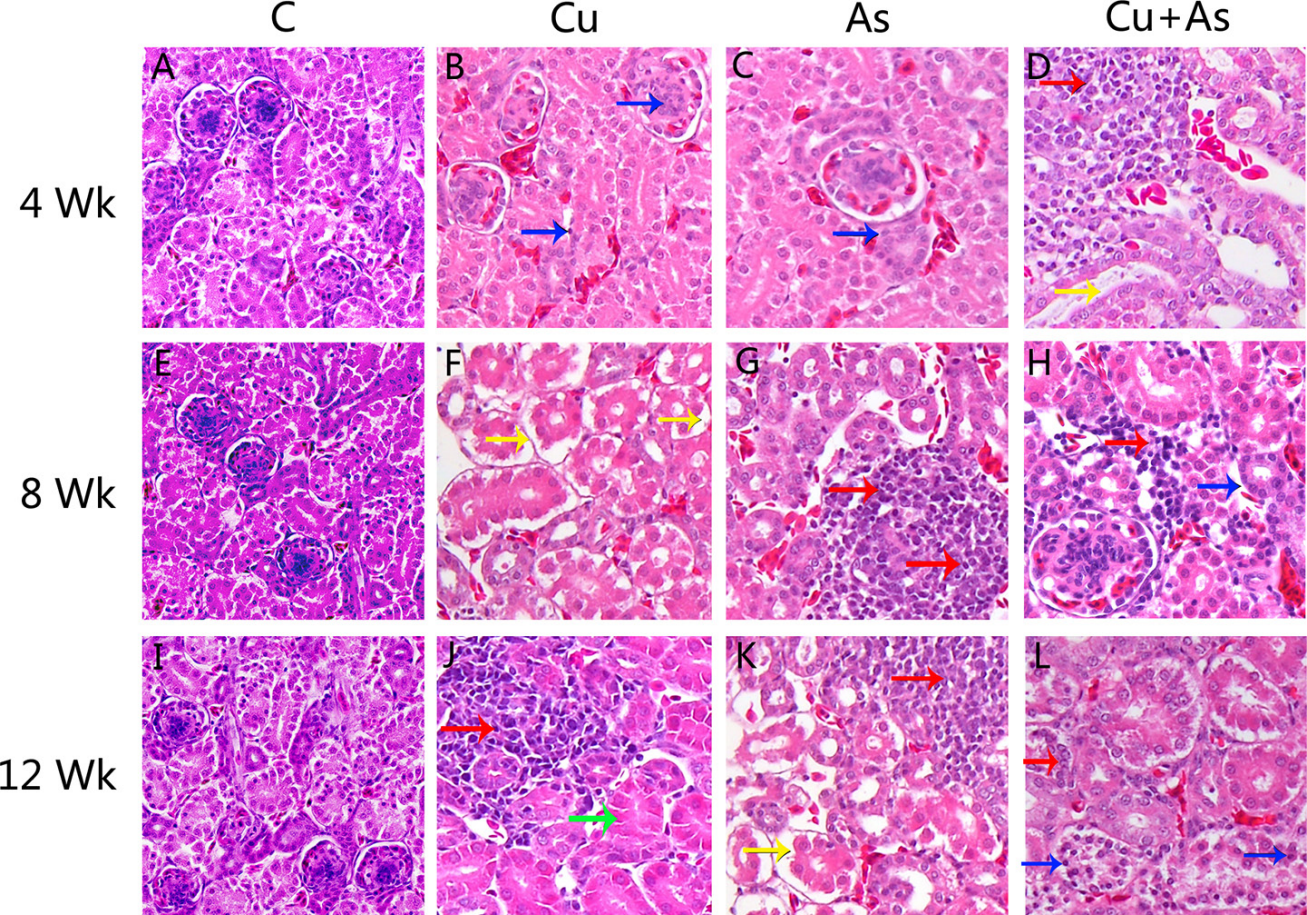
Histological changes during experiment (× 400) Arrows: red: lymphocytic infiltration, yellow: glomeruli swelling, blue: nuclear condensation of renal tubular epithelial cells, green: degeneration and necrosis of the tubular cells.

As shown in Figure 2, the degeneration and necrosis of the tubular cells, glomeruli swelling as well as the nuclear condensation of renal tubular epithelial cells and lymphocytic infiltration were observed in the experimental groups. Also, these histopathological lesions induced by Cu or/and As were changed in a time-dependent manner. The above lesions were not observed in the control group.

### Effects of Cu or/and As exposure on oxidative stress indicators of kidney

**Figure 3:**
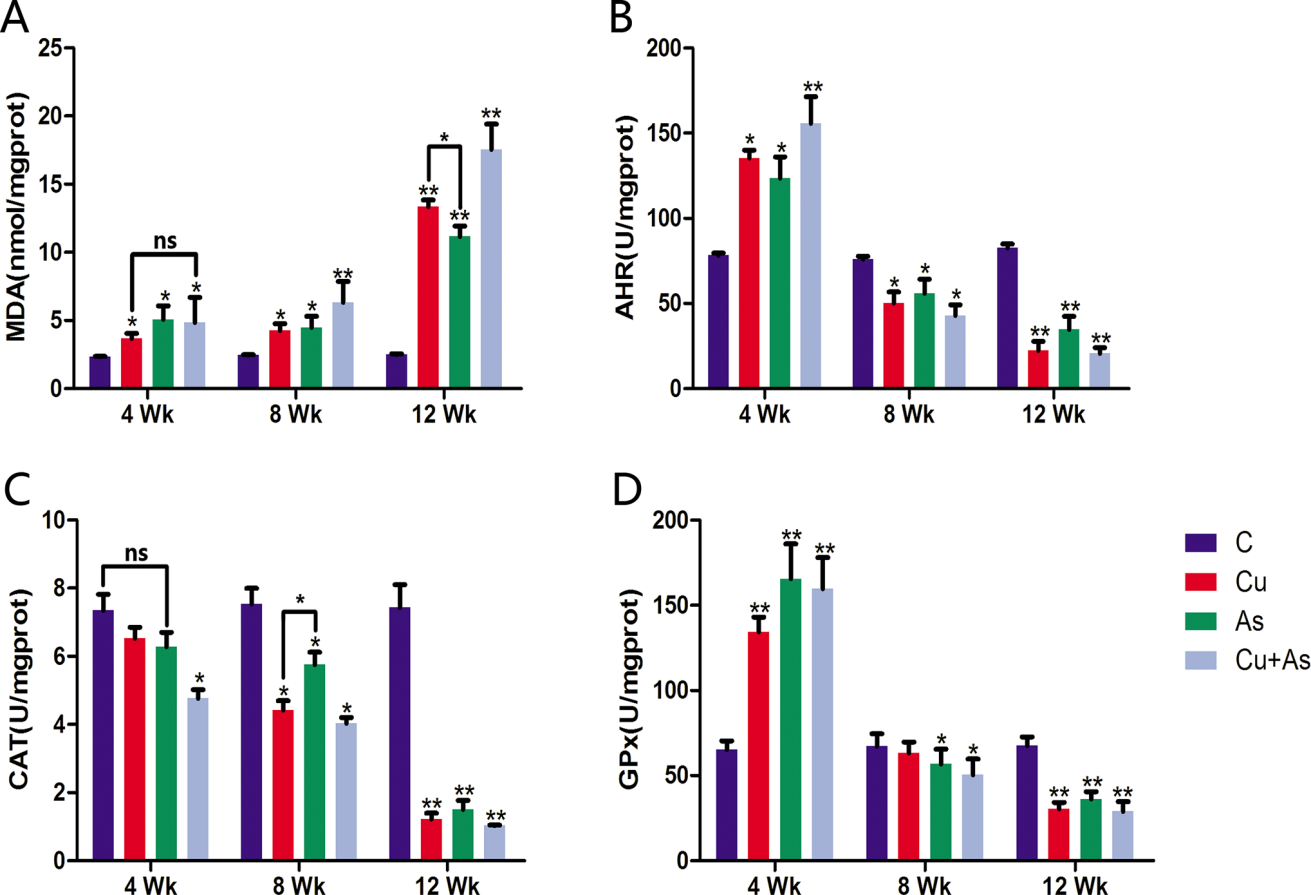
Changes in antioxidant system Each value is represented by mean ± SD. The asterisk indicates that there are significant differences (^*^P < 0.05 or ^**^P < 0.01) between the control group and the treatment groups at the same time point. ns, no significance.

Renal tubules are especially sensitive because of their possession of the common xenobiotic metabolizing enzymes and reabsorption function. Intracellular antioxidant enzymes protect biological macromolecules from oxidative stress induced organ pathophysiology. To investigate the status of the intracellular antioxidant defense machineries, we measured the activities of antioxidant enzymes, catalase (CAT) and glutathione peroxidase (GPx), the production of malondialdehyde (MDA), an end product of lipid peroxidation, and anti-hydroxy radical (AHR) ability. In the first 4 weeks, exposure to copper or arsenic alone significantly increased MDA (Figure 3A) production. However, coexposure group did not induce more severe lipid peroxidation compared to individual treatments (*P* > 0.05). Parallel, lower concentration reactive oxygen species in kidney were produced indicated by significant increase of AHR ability. The CAT activity declined pronouncedly in the co-exposed group as compared to individuals, which have no significant changes compared with control groups (*P* > 0.05). No additive effects of GPx activity in co-exposure were evident compared with Cu- or As-groups (*P* > 0.05). As antioxidant indicators, AHR and GPx show the organism's antioxidant capacity directly, exposure to Cu or/and As significantly increased kidney AHR ability and GPx activity compared to control group (*P* < 0.05 or *P* < 0.01). During the experiment after 8 weeks, treatment groups showed significant decreasing activities of enzymic antioxidants (CAT and GPx) along with inhibited AHR ability and increased MDA contents time-dependently in kidney (*P* < 0.05). Furthermore, we also observed more increased contents of MDA in Cu + As than Cu/As administration.

### Effects of Cu or/and As exposure on the mRNA and protein levels of inflammatory-related genes

**Figure 4:**
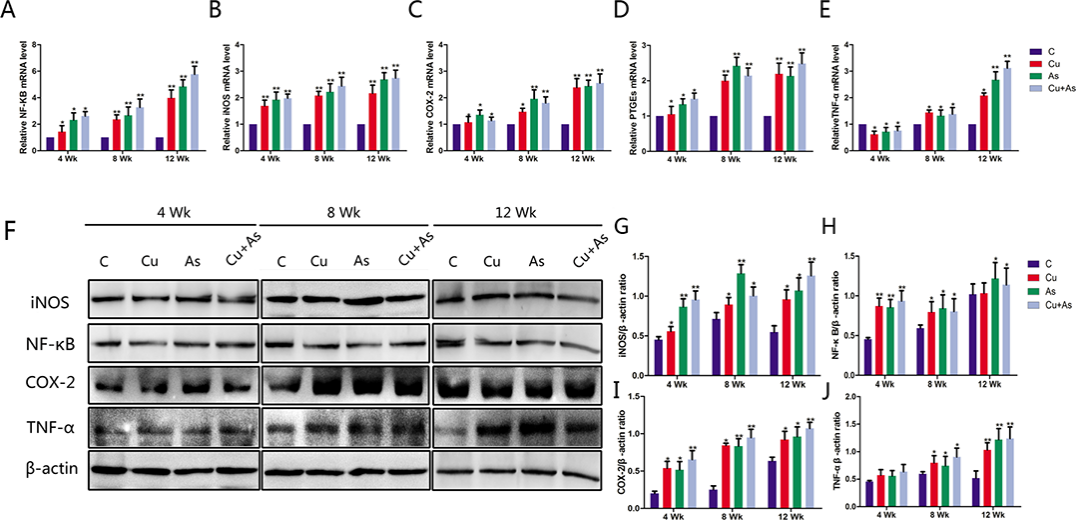
Changes in mRNA and protein levels of imflammation-related genes during experiment Each value is represented by mean ± SD. The asterisk indicates that there are significant differences (^*^P < 0.05 or ^**^P < 0.01) between the control group and the treatment groups at the same time point.

The effects of Cu or/and As on the mRNA levels of inflammatory-related genes including NF-κB, iNOS, COX-2, PTGEs and TNF-α were shown in Figure 4A–4E, which appeared a time-dependent fashion compared with the control group (*P* < 0.05 or *P* < 0.01). To directly investigate Cu and As-induced inflammation, the protein expression of inflammatory genes NF-κB, iNOS, COX-2 and TNF-α were assessed in the kidney homogenates, which showed similar expression pattern with their mRNA levels, respectively (Figure 4F–4H).

### Effects of Cu or/and As exposure on the cytokines levels

**Figure 5:**
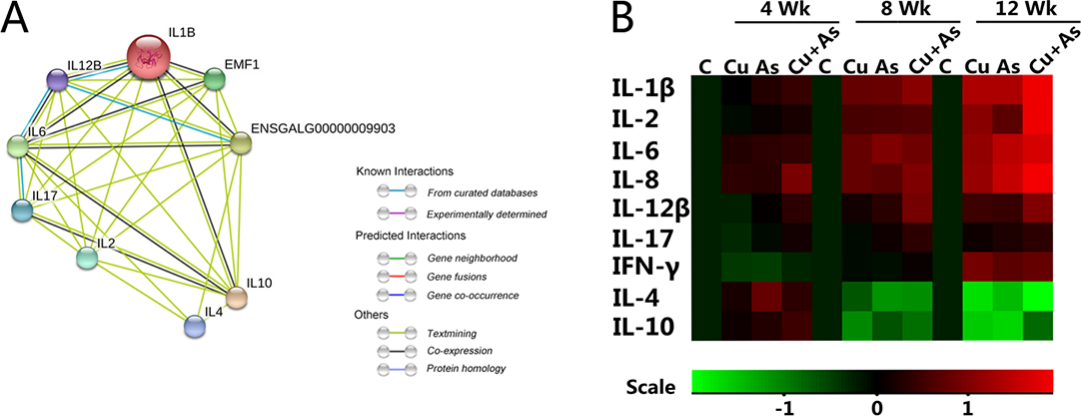
Protein network analysis and mRNA levels of immune-related genes (**A**) Protein network of proteins regulated between immune-related genes. (**B**) The heatmap of mRNA expression levels of immune-related genes. Values were expressed as mean ± SD. Symbol for the signifcance of differences between the transport group and control group: ^*^P < 0.05, ^**^P < 0.01. The mRNA expression levels of genes transcription are shown using the indicated pseudo color scale from -1 (green) to +1 (red) relative to values for control group. The color scale represents the relative mRNA expression levels, with red indicating up-regulated genes, green indicating down-regulated genes.

Multiple inflammatory cytokines were widely contact, and we researched the relationship of their coding genes through string network analysis. In Figure 5A, we studied 9 genes coding nine inflammatory cytokines. The predicted protein-protein interaction network showed that there might be a functional link between those proteins of genes we observed, some of which have been supported by other studies. Differently colored lines between genes present different meaning as labeled. The IL-1β (IL-1B) is co-expressed with genes of IL-6, IL-8 (EMF1), IL-10, IL-12β (IL-12B) and IFN-γ (ENSGALG00000009903). Most of cytokines were related with others, only IL-10 can mine text with all the other cytokines. It might function as the local immune controller or even inducer. To shed light upon this, a heat map showed the mRNA level of inflammatory cytokines in all groups (Figure 5B), its standard deviation (SD) was shown in Supplementary Table 1. These results revealed that although the level of IL-12β, IL-17 and IFN-γ have some tendencies to be inhibited in the first 4 weeks, a strong rebound was also detected after then. Other inflammatory cytokines, including IL-1β, IL-2, IL-6 and IL-8 showed statistical elevations in all weeks. Contrarily, the mRNA levels of IL-4, IL-10 displayed increase in 4 weeks then decrease during 8 weeks and 12 weeks time-dependently in kidney tissue suffered from Cu or/and As.

### Effects of Cu or/and As exposure on the HSPs production

**Figure 6:**
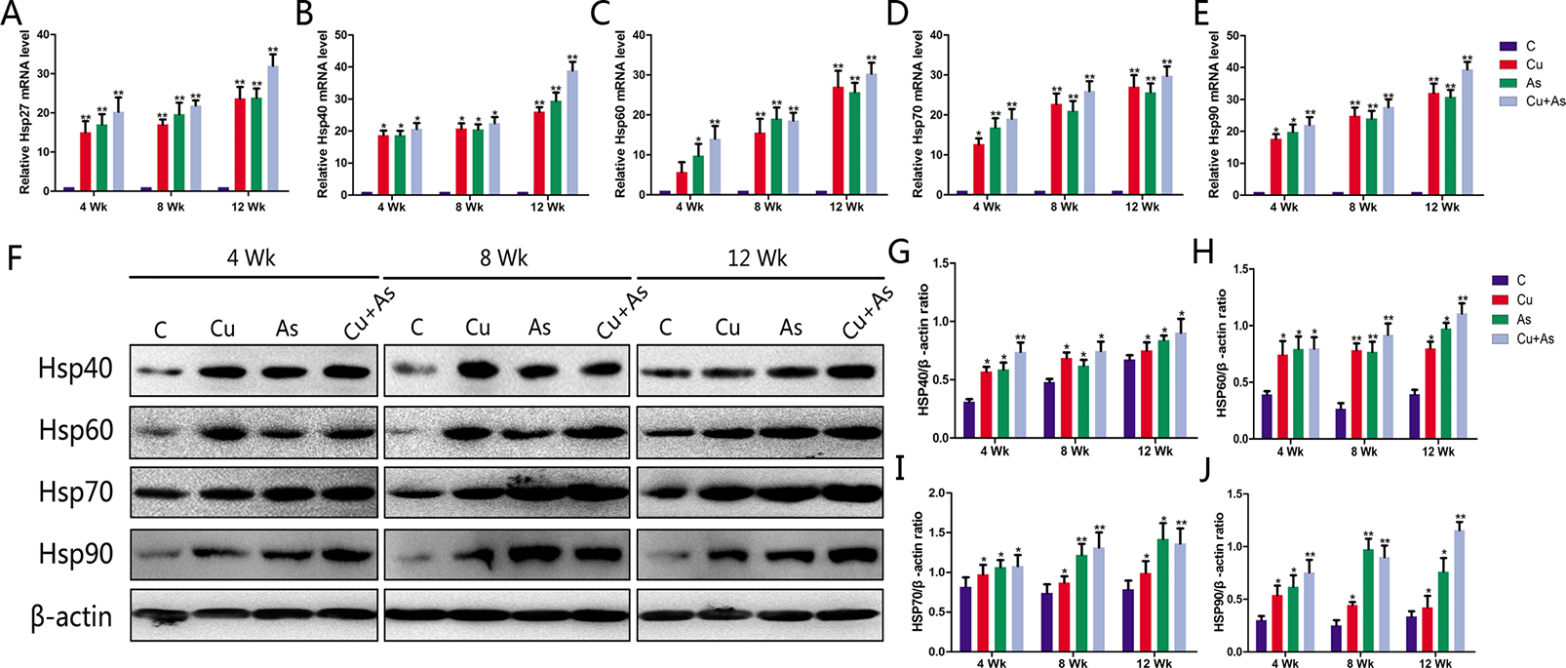
Changes in mRNA and protein levels of HSP-related genes during experiment Each value is represented by mean ± SD. The asterisk indicates that there are significant differences (^*^P < 0.05 or ^**^P < 0.01) between the control group and the treatment groups at the same time point.

The effects of Cu or/and As on the mRNA levels of HSP27/40/60/70/90 in the kidney tissues with different exposure time to 12 weeks were displayed in Figure 6A–6E. Cu or/and As exposure produced an up-regulation in the transcription of HSPs compared with control in the time-dependent manner (*P* < 0.05). The protein levels of HSP40/60/70/90 in the kidney tissues of chickens examined by Western blotting were shown in Figure 6F–6J. As expected, consistent with the transcription status of themselves, Cu or/and As exposure also significantly enhanced the protein level of HSPs (*P* < 0.05) in general comparing with the corresponding control groups during the experimental period.

### Correlation analysis of all indicators

Pearson's r correlation coefficient analysis (Supplementary Table 2) indicated significant positive correlations among NF-κB and indicators in inflammatory response and oxidative stress (at the 0.01 level). There were significant negative correlations between Th1 and Th2-secreted cytokines (at the 0.01 level). The results in Supplementary Table 2 exhibited a highly positive correlation both in single biological progress and conjoint analysis, which described the relationships among these factors and clearly revealed significant correlations among antioxidant factors and cytokines and HSPs.

### Principal component analysis

**Figure 7:**
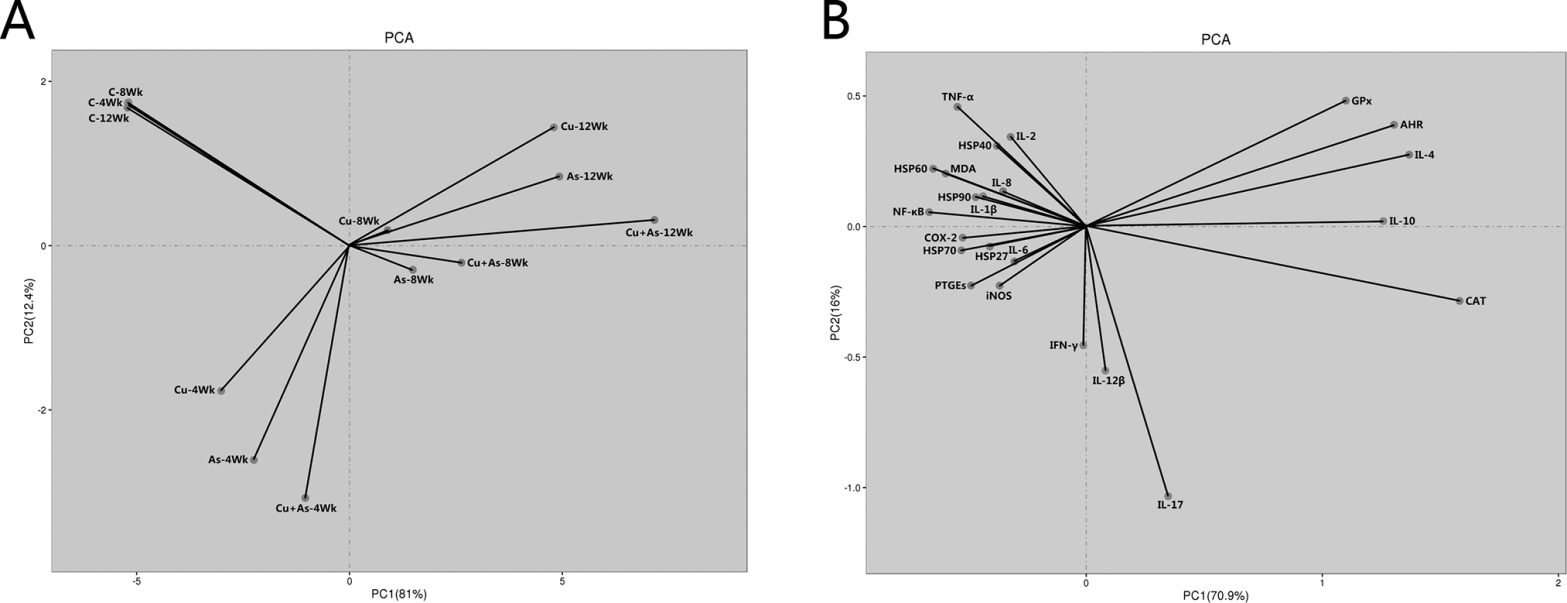
Ordination diagram of PCA for the parameters that were measured in the chicken kidney (**A**) Ordination diagram of PCA of groups measured in chicken kidney overexposed to CuSO4 or/and As2O3. (**B**) Ordination diagram of PCA of parameters measured in chicken kidney overexposed to CuSO4 or/and As2O3.

The results of the PCA were shown in Table 1 and Figure 7B. Parameter determination was based on ordination plots, corresponding to the first and second principal components as 70.9% and 16%, respectively. In addition, among these inflammatory indicators, AHR, CAT and GPx had clearly opposite relationships with MDA in PC1. Moreover, similar relationships were also indicated between IL-4, IL-10 and other Th1 cytokines.

**Table 1:** The correlation coefficients of the two principal components in chicken kidney

Element	PC1	PC2
MDA	0.206367	0.066492
AHR	−0.15304	−0.41548
CAT	−0.21999	−0.06447
GPx	−0.11643	−0.50427
NF-κB	0.229529	0.018326
iNOS	0.205378	−0.24978
COX-2	0.2252	−0.0755
PTGEs	0.215269	−0.13516
TNF-α	0.224171	0.060568
IL-1β	0.231089	0.001442
IL-2	0.221757	0.02478
IL-6	0.228181	−0.04205
IL-8	0.223719	−0.10588
IL-12β	0.208416	−0.06955
IL-17	0.203524	−0.09036
IFN-γ	0.197942	0.246631
IL-4	−0.18899	−0.31946
IL-10	−0.16664	−0.34724
HSP27	0.2075	−0.25473
HSP40	0.215062	−0.19502
HSP60	0.229454	−0.02839
HSP70	0.21741	−0.17437
HSP90	0.220051	−0.17308
Explained variance (%)	81.8%	17.2%

## DISCUSSION

Both arsenic and copper can accumulate in the urinary system. Arsenic exposure induces copper retention in kidney of rats and guinea pigs time- and dose-dependently [27]. However, what we find tremendously fascinating is that, the liver, another arsenic target organ, accumulates arsenic but the copper level is not affected [28]. These studies suggested that a functional relationship between copper and arsenic with respect to their distribution in the kidney may exist. Co-exposure to copper and arsenic may lead to more complicated adverse health effects than exposure alone. Thus, potential deleterious effects of concurrent subchronic exposure to copper and arsenic through diet were evaluated in kidney of chicken by measuring a range of parameters and histopathological examination.

Renal tubular cell survivability in the circulation depends on certain factors that affect their mechanical behavior, not least, the peroxidation of membrane phospholipids. This not only alters the lipid milieu and structural and functional integrity of cell membranes but also affects the activity of various membrane-bound enzymes. The toxicity of copper [4, 11] and arsenic [10] has been unambiguously demonstrated in a variety of experimental and epidemiological studies by disordering antioxidant enzymes and MDA production. During the first 4 weeks, the copper–arsenic-induced increase in MDA indicated generation of free radicals and subsequent oxidative stress-induced structural and functional changes in kidney. However, its content in the co-exposure group was not significantly different from that induced by either of the agents (*P* > 0.05), it seems that there was no appreciable interaction between them. On the other hand, a decrease in CAT activity by the coexposure was statistically less than that was observed with the individual compounds, indicating some additive interaction. Opposite additive interaction was also evident with AHR ability. Thus, it appears that the impact of the coexposure was too parameter-specific to generalize a specific type. Parallel activation of the antioxidant defense system of the cell reduces ROS generation. CAT and GPx constitutes the first line of cellular antioxidant defense system by scavenging free radicals [29]. The increased AHR ability is an indication of more production of ROS and dismutation to H_2_O_2_. To protect cells from peroxidation, activated cytosolic GPx performs the detoxification of ROS and generation of electrophiles, and leading more reduction of H_2_O_2_ to water [30]. The sharp rised enzymatic activities following Cu/As feeding can be due to adaptation on part of this system to counteract oxidative stress. However, the increase in MDA indicates these detoxification mechanisms cannot offset the exceeded production of free radicals. At individual treatment, there was no observable difference in CAT activity between control and treated chickens (*P* > 0.05). We believe that this is because control cells have produced sufficient CAT to mask any Cu or As-induced free radicals by 4 weeks of treatment. The decreased activity of CAT in co-exposure group might be due to inhibition by overproduced free radicals, impling Fenton-reaction-mediated conversion of more H_2_O_2_ to the ultimate toxicant, the HO^-^ [31]. Compared to arsenic, chickens suffered copper-diet exerted a low level of oxidant stress evidenced by the magnitude of MDA ranged by 56.84%, while 117.09% in arsenic-diet group. Moreover, co-exposure even showed a considerable antagonist to oxidative damage. It may be due to, firstly, the Fenton reaction can chelate Cu to limit its effects in free radical formation [32]. What's more, Cu-dependent transcription factors can regulate the synthesis of proteins by the transcription of specific genes encoding Cu/Zn superoxide dismutase, CAT and proteins related to the cellular storage of Cu [33]. Similar case was found in previous literature, which suggests that Cu supplementation (70 mg/L for 21 days) may have a protective effect against the Cd-induced oxidative stress in liver, kidney and placental tissues of rats [34]. Nevertheless, MDA was increased in all the three treatments, but CAT was decreased in coexposure group, showing MDA might relate more to the overproduction of free radicals rather than suppression of antioxidants. Hence, parameters detected in the first 4 weeks cannot identify the contribution of such changes to the development of certain clinical conditions in chickens.

However, after subchronic insult of Cu/As for 8 weeks and longer, excessive oxidative stress elevated production of ROS, damaged membrane structures heavily, leading the failure of adaptive mechanism, as evidenced by soaring content of lipid peroxidation and declining enzymes activities. These results parallel those reported in goldfishes [35], in which high intakes of Cu increases the concentration of MDA in embryos and larvae, while CAT activities were inhibited. Another study modeled by chicken [8] is also in line with our results, showing depressing activities of GPx in association with excessive accumulation of arsenic in the spleen. Although GPx and CAT catalyze the same substrate H_2_O_2_, the former cycle is a major protective mechanism against low levels of oxidant stress, whereas CAT, against the severe [36]. The obtained results showed in the middle and later period of present study, the magnitude of MDA ranged by multiples, indicating a high level of oxidant stress, and CAT activity was decreased markedly by 24–86%, suggesting that the CAT redox cycle might exert more important role than GPx redox cycle, which reduction rate teeters 1–57%. Compared to arsenic, copper administration leads to higher content of MDA and lower contents of antioxidants, implying that Cu acts as a stronger oxidative stress inducer. On the other hand, compared to the effects of either of the individual agents or control group, the effects produced by coexposure on these parameters were pronouncedly altered. Previous studies also found that binary mixtures of As (50 μM) with Cd or Pb caused a significant increase in the cytotoxicity to HepG2 cells compared to those of individual metals characterized by elevated ROS production and decreased antioxidant enzymes activity [37]. In terms of oxidative stress, adverse effects got aggravated during Cu/As combination administration for subchronic. As the increase in MDA was associated with increase in ROS level and decreases in antioxidants, it is a potential indicator of renal peroxidative damage as well as redox homeostasis.

The present observation showed that dietary exposure of chickens to Cu/As enhanced production of NO evidenced by elevated levels of iNOS, which may be explained by generation of free radicals [38]. In fact, as electrophile, ROS initiate the lipid peroxidation process which do overwhelm the antioxidant defense system in kidney, then renal injury cascaded by oxidative stress occurs. H&E results showed that subchronic exposure to Cu/As induced nuclear condensation of renal tubular epithelial cells and lymphocytic infiltration time-dependently. On the basis of the essential involvement of NF-κB in tissue injury, we supposed that Cu/As-induced potent inflammatory responses were due to the activation of NF-κB. Results displayed that the mRNA and protein expression levels of TNF-α, PTGEs, COX-2 were increased in treatment groups in individual/coexposure groups (Figure 4), which were consistent with previous study [8]. We also found that the mRNA and protein expression of NF-κB and iNOS were increased the activity of iNOS, supporting our hypothesis. The inflammatory cell infiltration may also account for the increased production of various inflammatory cytokines, especially interleukins. Interleukins, as important indicators of the inflammatory response, have attracted much attention in recent years. Effects of metal exposure on interleukins, such as manganese on IL-1β and IL-2 [39], nickel on the neurotrophic IL-6 [40], selenium on neutrophil chemotactic IL-8 and IL-17 [41] so forth, have been widely described. Due to the postulation about a significant role of cytokines in Cu/As -induced kidney injury, we detected the mRNA levels of some proinflammatory cytokines, including IL-1β, IL-2, IL-6, IL-8, IL-12β, IL-17, IFN-γ, and anti-inflammatory cytokines, including IL-4 and IL-10. According to the secretory cells, some of them can also be divided into Th1 cells-secreted cytokines, namely IL-2, IL-12β, IFN-γ as well as Th2 cells-secreted cytokines, namely IL-4, IL-10. Results displayed that the mRNA levels of proinflammatory cytokines have sustained increases in treatment groups, which might be due to lipid peroxidation results in decreased glomerular filtration rate, making excessive of cytokines recruitment. Similary, the mRNA expression levels of anti-inflammatory cytokines had a first increase in the first 4 weeks, while following a decreasing tendency in subchronic Cu or/and As poisoning, which displayed irrepressible immune disorders and inflammatory lesions. These results also indicated Th2 response is suppressed as shown by diminished IL-4 and IL-10 in kidney after Cu or/and As exposure, and enhancement of Th1 response is marked by increased in IFN-γ, IL-2 and IL-12β. These results were coincident with previous study [42], indicating the “Th1/Th2 drift” has a shift to Th1, which hints at the destruction of the dynamic balance of cytokine network.

As sophisticated stress response mechanisms to maintain or re-establish cellular homeostasis, heat shock proteins unselectively bind to hydrophobic protein sequences liberated by denaturation and keep protein homeostasis from cellular and environmental stress factors as molecular chaperones [43]. Exposure to arsenic stimulation greatly increases the phosphorylation levels of Hsp27/40/60/70/90, inhibiting protein phosphatase activity in human urothelial cells [44], Bursa of Fabricius, spleen and thymus of chickens [25]. In fact, facing copper, the redox-active metal, HSPs also display an intransigent spirit, their increased levels in response to copper ions occurred in a dose-dependent manner [45]. In the present study, the expression of HSPs mRNA and protein were increased by 5 to 40 folds in the treatment group compared with the control group. The expression differences of HSPs between the treated group and the combined treatment group were analyzed to estimate the effect of Cu and As each other. It showed that Cu and As boosted the occurring of oxidative damage mutually, leading significantly elevated levels of HSPs. Actually, the expression of proinflammatory cytokine, IL-1β, IL-6 and TNF-α mRNAs has been associated with the expression of Hsp60, Hsp47 and Hsp70 mRNAs, respectively [46], which hints the severe inflammation. From the arsenicals studied, arsenic is a great inducer of HSPs in several organs with a rapid dose-dependent answer [47], thus the stress response appears to be a viable biomarker of sublethal toxicity as a result of a single exposure to arsenic. In addition, the over-expressions of HSP60/70 were also observed in the intertidal copepod [45] and human embryonic carcinoma cell line [48] exposure to different concentrations of copper. As molecular chaperones, HSP90 and HSP60 might play important roles in protecting the gastrointestinal tracts from As_2_O_3_-induced cell damage [9]. As expected, these data demonstrated that HSPs were vital protective proteins in the kidney tissues of chickens against subchronic Cu or/and As poisoning-cascaded oxidative stress and inflammation. However, how long this protection will last needs further study.

**Figure 8:**
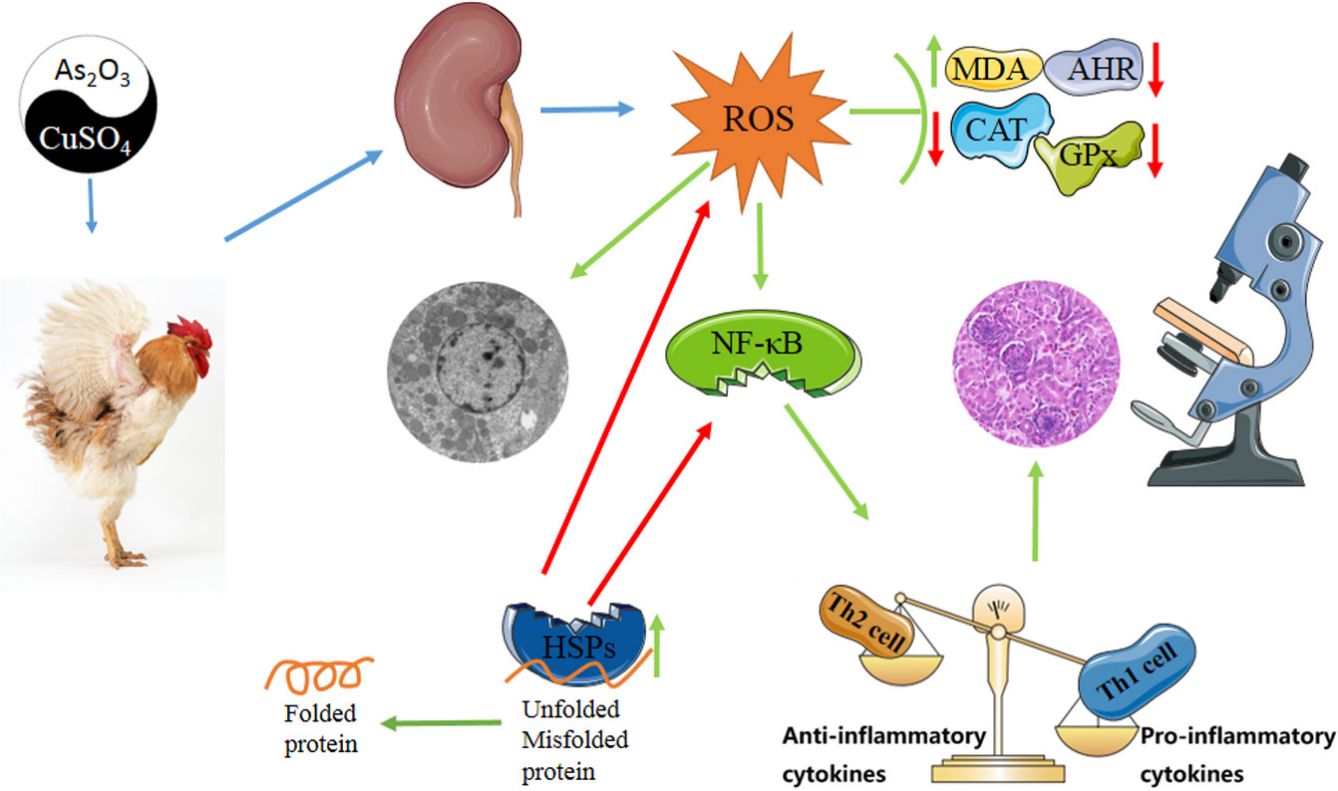
Diagram depicts the toxic effect of CuSO4 and As2O3 on chicken kidney “Th1/Th2 drift” has a shift to Th1 because of damaged immune defense system in kidney of chickens suffering from subchronic Cu or/and As poisoning, and oxidative stress as well as subsequent inflammatory is a crucial driver during exposure. HSPs maintain cellular homeostasis by assisting in the correct folding of nascent and stress-accumulated misfolded proteins. Green arrows mean promotion or up-regulation, red arrows mean inhibition or down-regulation.

In conclusion, sub-chronic exposure to Cu or/and As induced nephrotoxicity in chickens. This exposure makes a destruction in antioxidant system and immune system synergistically. Noteworthy, co-exposure to Cu and As showed a considerable antagonist to oxidative damage in the early stage. Meanwhile, mRNA levels and protein expressions of HSP27/40/60/70/90 were also increased, which may play a renoprotective function against tissues damage caused by stress response (Figure 8).

## MATERIALS AND METHODS

### Animals and treatment

Seventy-two male Hy-line chickens (one-day-old; 50–55 g; purchased by Weiwei Co. Ltd., China) were housed in the Institutional Animal Care and Use Committee of Northeast Forestry University (Harbin, China) (approval no. UT-31; 20 June 2014). Chickens were randomly divided into four groups (18 chickens per group), including a control group: basal diet, three treatment groups: 300 mg/kg of CuSO_4_ [49] or 30 mg/kg of As_2_O_3_ [8]-mixed feed group or similarly coexposed to these in the same dose levels. The composition of the diet was: Maize, grains 421 g/kg; wheat, grains 120 g/kg; full fat soy 180 g/kg; pea 100 g/kg; wheat bran 80 g/kg; limestone 80 g/kg; dicalcium phosphate 15 g/kg and sodium chloride 4 g/kg. This diet met the minimum requirements for energy and nutrients for chicken and without influencing results [50]. The experimental animals were housed in an environmentally controlled room with a temperature of 23 ± 2°C and a relative humidity within the range of 40–70%. The air was changed 10–15 times per hour. The light was set for a 12 h light and dark cycle. All chickens were examined for clinical signs of ill health and observed during the experiment. Animal studies, including animal care and all experimental procedures, were in accordance with the Animal Welfare Guidelines of Northeast Forestry University and the inhouse guidelines of the Institutional Animal Care and Use Committee in Harbin, China. Animal experiment protocols were reviewed and approved by the Animal Care, Use and Ethics Committee at Northeast Forestry University (approval no. UT-31; 20 June 2014). Six chickens in each group were selected randomly at 4, 8 and 12 weeks of the experiment and euthanized with sodium pentobarbital (30 mg/kg BW). The kidney tissues were quickly excised and blotted then rinsed with ice-cold 0.9% NaCl solution, frozen immediately in liquid nitrogen, and stored at −80°C until required.

### Histological and ultrastructural observations

Kidney tissue specimens (size, 1.0 mm^3^) from the typical sample of control group, Cu group, As group and Cu + As group at every time points were rapidly fixed with 2.5% glutaraldehyde phosphate buffer saline (v/v, pH 7.2), postfixed in 1% osmium tetroxide (v/v), and stained with 4.8% uranyl acetate. Then, samples were dehydrated in a graded series of ethanol and embedded in Epon. Ultrathin (less than or equal to 90 nm) sections were cut, mounted on coated copper grids, washed in propylene oxide, impregnated with epoxy resins and post-stained with uranyl acetate and lead citrate. The specimens were observed with microscopy. Microphotographs were taken with a transmission electron microscope (GEM-1200ES, Japan). For light microscopy, small pieces of kidney tissues (size, 1.0 mm^3^) were fixed in 4% paraformaldehyde, dehydrated in ethanol and embedded in paraffin. Serial slices at 5 μm thickness were prepared and stained with haematoxylin and eosin (H&E), and examined by light microscopy.

### Determination of antioxidant system

The kidney tissues taken at different time-points were homogenized in 0.9% NaCl solution and centrifuged at 3000 × g for 5 min. The supernatants were collected to determine the antioxidant function. Brief procedures were listed as the following. The activities of CAT and GPx, MDA contents and AHR ability were measured six copies using the corresponding detection kits (Nanjing Jiancheng Bioengineering Institute, China) according to the manufacture's protocol. Absorbance of the supernatant was measured at 405, 412, 532 and 600 nm. The results of spectrophotometric analysis were expressed as international units in nmol per protein.

### Quantitative real-time PCR of Cytokines and HSPs

Total RNA was isolated from the kidney tissue samples (50 mg tissue; *n* = 6/group) of chickens using RNAiso Plus reagent (Takara, Japan) according to the manufacturer's instructions. The concentrations and purity of the total RNA were determined by spectrophotometer (Ultrospec 1100 *pro,* Amersham Biosciences, China) at OD_260/280_ nm. Total RNA (50 μg) was reverse transcribed into complementary DNA (cDNA) using the PrimeScript^TM^ RT Reagent Kit (Takara, Japan). Synthesized cDNA was diluted ten times with sterile water and stored at −80°C before use.

Specific primers used for amplification were designed based on known chicken sequences (Table 2) using Primer Premier software (PREMIER Biosoft International, USA). The relative mRNA levels of cytokines- and HSPs-related genes were performed on a LightCycler^®^ 480 (Roche, Switzerland) Real-Time PCR System (Hangzhou, China) and determined with the FastStart Universal SYBR Green Master reagents (Roche, Switzerland). The detailed conditions of PCR protocol and calculation method of each gene relative mRNA abundance are indicated in our previous research [8].

**Table 2:** A list of primers in qRT-PCR analysis of mRNA expression of the target genes

Genes	GenBank accession	Primer sequence(5’→3’)	Product size
NF-κB	NM205134	Forward: TCAACGCAGGACCTAAAGACAT	162 bp
		Reverse: GCAGATAGCCAAGTTCAGGATG	
TNF-α	NM204267	Forward: GCCCTTCCTGTAACCAGATG	71 bp
		Reverse: ACACGACAGCCAAGTCAACG	
PTGES	NM001194983	Forward: GTTCCTGTCATTCGCCTTCTAC	115 bp
		Reverse: CGCATCCTCTGGGTTAGCA	
COX-2	NM001167718	Forward: TGTCCTTTCACTGCTTTCCAT	84 bp
		Reverse: TTCCATTGCTGTGTTTGAGGT	
iNOS	NM204961	Forward: CCTGGAGGTCCTGGAAGAGT	82 bp
		Reverse: CCTGGGTTTCAGAAGTGGC	
IL-1β	NM204524	Forward: CAGCAGCCTCAGCGAAGAG	86 bp
		Reverse: CTGTGGTGTGCTCAGAATCCA	
IL-2	AF033563	Forward: TTCAAAATATCGAAAAGAACCTCAAG	51 bp
		Reverse: CGGTGTGATTTAGACCCGTAAGAC	
IL-6	NM204628	Forward: AAATCCCTCCTCGCCAATCT	106 bp
		Reverse: CCCTCACGGTCTTCTCCATAA A	
IL-8	NM205498	Forward: GGCTTGCTAGGGGAAATGA	199 bp
		Reverse: AGCTGACTCTGACTAGGA AACTGT	
IL-12β	NM213571	Forward: TGTCTCACCTGCTATTTGCCTTAC	87 bp
		Reverse: CATACACATTCTCTCTAAGTTTCCACTGT	
IL-17	AY744450	Forward: CATGTTGTCAGCCAGCATTTCT	107bp
		Reverse: CATCTTTTTGGGTTAGGCATCC	
IFN-γ	GQ246226	Forward: GTGAAGAAGGTGAAAGATATCATGGA	71 bp
		Reverse: GCTTTGCGCTGGATTCTCA	
IL-4	AJ621249	Forward: GTGCCCACGCTGTGCTTAC	82 bp
		Reverse: AGGAAACCTCTCCCTGGATGTC	
IL-10	AJ621614	Forward: CGCTGTCACCGCTTCTTCA	88bp
		Reverse: TCCCGTTCTCATCCATCTTCTC	
HSP27	NM205290	Forward: ACACGAGGAGAAACAGGATGAG	158 bp
		Reverse: ACTGGATGGCTGGCTTGG	
HSP40	NM001199325	Forward: GGGCATTCAACAGCATAGA	151 bp
		Reverse: TTCACATCCCCAAGTTTAGG	
HSP60	NM001012916	Forward: AGCCAAAGGGCAGAAATG	208 bp
		Reverse: TACAGCAACAACCTGAAGACC	
HSP70	NM001006685	Forward: CGGGCAAGTTTGACCTAA	250bp
		Reverse: TTGGCTCCCACCCTATCTCT	
HSP90	NM001109785	Forward: TCCTGTCCTGGCTTTAGTTT	143 bp
		Reverse: AGGTGGCATCTCCTCGGT	
β-actin	NM205518	Forward: CCGCTCTATGAAGGCTACGC	128 bp
		Reverse: CTCTCGGCTGTGGTGGTGAA	

### Western blotting analysis for HSP40, HSP60, HSP70, HSP90, TNF-α, COX-2, iNOS and NF-κB

Protein samples from kidney tissues were extracted using SDS Lysis Buffer and quantitied with Enhanced BCA Protein Assay Kit (Beyotime, China). After SDS-PAGE, proteins were transferred to the polyvinylidene fluoride (PVDF) membrane from gel without staining, HSP40 (1:10000, Abcam, UK), HSP60/90/TNF-α/COX-2/β-actin (1:1000, Proteintech, China), HSP70/iNOS (1:500, Bioss Antibodies, China), NF-κB (1:500, WanleiBio, China) were used as the primary antibodies, and specific reaction products were detected with horseradish peroxidase (HRP)-conjugated secondary antibody. The conditions for Western blotting have been described previously [51].

### Statistical analysis

Statistical analyses of all data were performed using SPSS for Windows (version 21.0; SPSS Inc., Chicago, IL) and assessed with a one-way analysis of variance (ANOVA). All values were expressed as the mean ± SD. Differences between the means of the control group and experimental groups were considered to be significant at ^*^P < 0.05 or ^**^P < 0.01. Correlation analysis was used to determine the relationship between individual variations using GraphPad Software Prism 5 (version 5.01, GraphPad Software, Inc., La Jolla, USA). PCA and heat map were performed using the OmicShare tools, a free online platform for data analysis (www.omicshare.com/tools). Equally, protein-protein interaction network of genes was constructed via STRING 10 (https://string-db.org/).

## SUPPLEMENTARY MATERIALS TABLES





## Abbreviations

As_2_O_3_: arsenic trioxide
Cu: Copper
ROS: reactive oxygen species
NF-κB: nucleic factor κB
iNOS: inducible nitric oxide synthase
COX-2: cyclooxygenase-2
PTGEs: prostaglandin E synthase
TNF-α: tumor necrosis factor-α
IL: interleukin
HSPs: Heat shock proteins
PCA: principal components analysis
CAT: catalase
GPx: glutathione peroxidase
MDA: malondialdehyde
AHR: anti-hydroxy radical
IFN: interferon
