# Supplementary material for: Copper or/and arsenic induce oxidative stress-cascaded, nuclear factor kappa B-dependent inflammation and immune imbalance, trigging heat shock response in the kidney of chicken
Source: Oncotarget. 2017 Oct 3;8(58):98103–16. doi: 10.18632/oncotarget.21463 (PMC5716717; doi:10.18632/oncotarget.21463)
Supplement: Supplementary file 1 [file oncotarget-08-98103-s001.pdf]

Copper or/and arsenic induce oxidative stress-cascaded, nuclear factor kappa B-dependent inflammation and immune imbalance, trigging heat shock response in the kidney of chicken

SUPPLEMENTARY MATERIALS

Supplementary Table 1: Standard error of the mean of the heat map in Figure 5B

	4 Wk				8 Wk				12 Wk			
	C	Cu	As	Cu+As	C	Cu	As	Cu+As	C	Cu	As	Cu+As
IL-1β	0	0.136854	0.158085	0.167459	0	0.157224	0.148628	0.149890	0	0.193567	0.184654	0.195547
IL-2	0	0.127024	0.084624	0.175932	0	0.128435	0.118575	0.104847	0	0.111564	0.124568	0.119739
IL-6	0	0.153856	0.18563	0.298304	0	0.168872	0.178348	0.135839	0	0.164758	0.215367	0.224264
IL-8	0	0.294725	0.285624	0.337656	0	0.236286	0.258375	0.337656	0	0.244595	0.307532	0.307637
IL-12β	0	0.084702	0.109890	0.119982	0	0.077674	0.098743	0.113767	0	0.105767	0.115463	0.109756
IL-17	0	0.09342	0.112887	0.117453	0	0.136936	0.127908	0.135804	0	0.09	0.111668	0.104794
IFN-γ	0	0.073390	0.097892	0.072649	0	0.084342	0.098785	0.089764	0	0.097690	0.060542	0.195685
IL-4	0	0.103759	0.082645	0.109892	0	0.075645	0.083957	0.068593	0	0.074367	0.088448	0.085673
IL-10	0	0.115924	0.098792	0.087860	0	0.078630	0.084856	0.069758	0	0.075645	0.064563	0.065846

Supplementary Table 2: Pearson's r correlation coefficient matrix among indicators measured in the chicken kidney $^{*}P < 0.05$; $^{\#}P < 0.01$. See_Supplementary_Table 2
