# Supplementary material for: Copper or/and arsenic induce oxidative stress-cascaded, nuclear factor kappa B-dependent inflammation and immune imbalance, trigging heat shock response in the kidney of chicken
Source: Oncotarget. 2017 Oct 3;8(58):98103–16. doi: 10.18632/oncotarget.21463 (PMC5716717; doi:10.18632/oncotarget.21463)
Supplement: Supplementary file 2 [file oncotarget-08-98103-s002.docx]

**Table 2: Pearson’s r correlation coefficient matrix among indicators measured in the chicken kidney** **P* < 0.05; #*P* < 0.01

|  | MDA | AHR | CAT | GPx | NF-κB | iNOS | COX-2 | PTGEs | TNF-α | IL-1β | IL-2 | IL-6 | IL-8 | IL-12β | IL-17 | IFN-γ | IL-4 | IL-10 | HSP27 | HSP40 | HSP60 | HSP70 | HSP90 |
| --- | --- | --- | --- | --- | --- | --- | --- | --- | --- | --- | --- | --- | --- | --- | --- | --- | --- | --- | --- | --- | --- | --- | --- |
| MDA | 1.000 |  |  |  |  |  |  |  |  |  |  |  |  |  |  |  |  |  |  |  |  |  |  |
| AHR | -0.607# | 1.000 |  |  |  |  |  |  |  |  |  |  |  |  |  |  |  |  |  |  |  |  |  |
| CAT | -0.912# | 0.640# | 1.000 |  |  |  |  |  |  |  |  |  |  |  |  |  |  |  |  |  |  |  |  |
| GPx | -0.502* | 0.942# | 0.549# | 1.000 |  |  |  |  |  |  |  |  |  |  |  |  |  |  |  |  |  |  |  |
| NF-κB | 0.860# | -0.679# | -0.937# | -0.520* | 1.000 |  |  |  |  |  |  |  |  |  |  |  |  |  |  |  |  |  |  |
| iNOS | 0.680# | -0.312 | -0.773# | -0.097 | 0.884# | 1.000 |  |  |  |  |  |  |  |  |  |  |  |  |  |  |  |  |  |
| COX-2 | 0.840# | -0.560# | -0.886# | -0.372* | 0.981# | 0.934# | 1.000 |  |  |  |  |  |  |  |  |  |  |  |  |  |  |  |  |
| PTGEs | 0.695# | -0.504* | -0.801# | -0.297 | 0.928# | 0.952# | 0.953# | 1.000 |  |  |  |  |  |  |  |  |  |  |  |  |  |  |  |
| TNF-α | 0.928# | -0.688# | -0.944# | -0.565# | 0.958# | 0.810# | 0.927# | 0.838# | 1.000 |  |  |  |  |  |  |  |  |  |  |  |  |  |  |
| IL-1β | 0.895# | -0.671# | -0.942# | -0.505* | 0.982# | 0.878# | 0.962# | 0.926# | 0.965# | 1.000 |  |  |  |  |  |  |  |  |  |  |  |  |  |
| IL-2 | 0.922# | -0.668# | -0.902# | -0.523* | 0.918# | 0.796# | 0.901# | 0.866# | 0.944# | 0.970# | 1.000 |  |  |  |  |  |  |  |  |  |  |  |  |
| IL-6 | 0.854# | -0.603# | -0.910# | -0.441* | 0.983# | 0.920# | 0.983# | 0.945# | 0.966# | 0.978# | 0.933# | 1.000 |  |  |  |  |  |  |  |  |  |  |  |
| IL-8 | 0.871# | -0.477* | -0.936# | -0.333 | 0.941# | 0.917# | 0.939# | 0.902# | 0.942# | 0.962# | 0.922# | 0.964# | 1.000 |  |  |  |  |  |  |  |  |  |  |
| IL-12β | 0.769# | -0.553# | -0.819# | -0.376* | 0.861# | 0.826# | 0.838# | 0.858# | 0.814# | 0.914# | 0.881# | 0.851# | 0.888# | 1.000 |  |  |  |  |  |  |  |  |  |
| IL-17 | 0.718# | -0.506* | -0.796# | -0.332 | 0.853# | 0.826# | 0.832# | 0.854# | 0.796# | 0.886# | 0.822# | 0.844# | 0.884# | 0.970# | 1.000 |  |  |  |  |  |  |  |  |
| IFN-γ | 0.909# | -0.790# | -0.927# | -0.745# | 0.859# | 0.549# | 0.779# | 0.629# | 0.891# | 0.846# | 0.829# | 0.798# | 0.776# | 0.679# | 0.655# | 1.000 |  |  |  |  |  |  |  |
| IL-4 | -0.764# | 0.915# | 0.812# | 0.892# | -0.820# | -0.493* | -0.722# | -0.659# | -0.826# | -0.814# | -0.818# | -0.767# | -0.689# | -0.695# | -0.656# | -0.897# | 1.000 |  |  |  |  |  |  |
| IL-10 | -0.605# | 0.903# | 0.752# | 0.854# | -0.770# | -0.448* | -0.660# | -0.581# | -0.737# | -0.705# | -0.629# | -0.683# | -0.567# | -0.511* | -0.490* | -0.842# | 0.872# | 1.000 |  |  |  |  |  |
| HSP27 | 0.731# | -0.290 | -0.806# | -0.081 | 0.885# | 0.988# | 0.940# | 0.942# | 0.813# | 0.888# | 0.821# | 0.915# | 0.932# | 0.833# | 0.825# | 0.588# | -0.497* | -0.426* | 1.000 |  |  |  |  |
| HSP40 | 0.830# | -0.365* | -0.855# | -0.185 | 0.904# | 0.960# | 0.946# | 0.916# | 0.896# | 0.925# | 0.895# | 0.951# | 0.968# | 0.837# | 0.811# | 0.667# | -0.576# | -0.462* | 0.972# | 1.000 |  |  |  |
| HSP60 | 0.881# | -0.614# | -0.942# | -0.448* | 0.984# | 0.889# | 0.976# | 0.937# | 0.946# | 0.986# | 0.942# | 0.975# | 0.962# | 0.882# | 0.878# | 0.843# | -0.781# | -0.686# | 0.910# | 0.925# | 1.000 |  |  |
| HSP70 | 0.749# | -0.437* | -0.853# | -0.224 | 0.928# | 0.974# | 0.954# | 0.974# | 0.849# | 0.936# | 0.869# | 0.939# | 0.939# | 0.881# | 0.862# | 0.656# | -0.602# | -0.550# | 0.981# | 0.953# | 0.946# | 1.000 |  |
| HSP90 | 0.800# | -0.429* | -0.862# | -0.231 | 0.935# | 0.973# | 0.970# | 0.968# | 0.882# | 0.946# | 0.902# | 0.961# | 0.958# | 0.866# | 0.843# | 0.680# | -0.622# | -0.528# | 0.985# | 0.982# | 0.956# | 0.989# | 1.000 |
